# Respiratory effects on phase contrast imaging of the jugular vein

**DOI:** 10.1186/1532-429X-14-S1-W4

**Published:** 2012-02-01

**Authors:** Eric M Schrauben, Ashley G Anderson, Kevin Johnson, Aaron Field, Oliver Wieben

**Affiliations:** 1 Medical Physics, University of Wisconsin - Madison, Madison, WI, USA; 2 Department of Radiology, University of Wisconsin - Madison, Madison, WI, USA

## Summary

An analysis of the effect of respiratory function on MR flow measures of the internal jugular vein (IJV) is presented. A novel 2D radial acquisition and reconstruction method allows for retrospective gating to both the cardiac and respiratory cycle. In-vivo scans of human volunteers verify the efficacy of the algorithm, showing increased IJV flow during inspiration and decreased flow during expiration for each cardiac time frame.

## Background

The introduction of the CCSVI hypothesis in the diagnosis of MS has recently caused interest in intra- and extracranial venous flow measurements (1). Due to structural complexity and individual variations, flow studies in cerebrospinal veins using phase-contrast (PC) MR are rarely conducted (2). Though it has long been confirmed a source of variability in venous drainage to the heart (3), respiratory motion effects have been largely ignored in PC-MR, partially due to the difficulty and longer scan times of gating the respiratory and cardiac cycles. The purpose of this pilot study was to implement a dual-gated PC-MR sequence and investigate the effect of respiratory motion during free breathing on cerebrospinal venous flow.

## Methods

**Figure 1:**
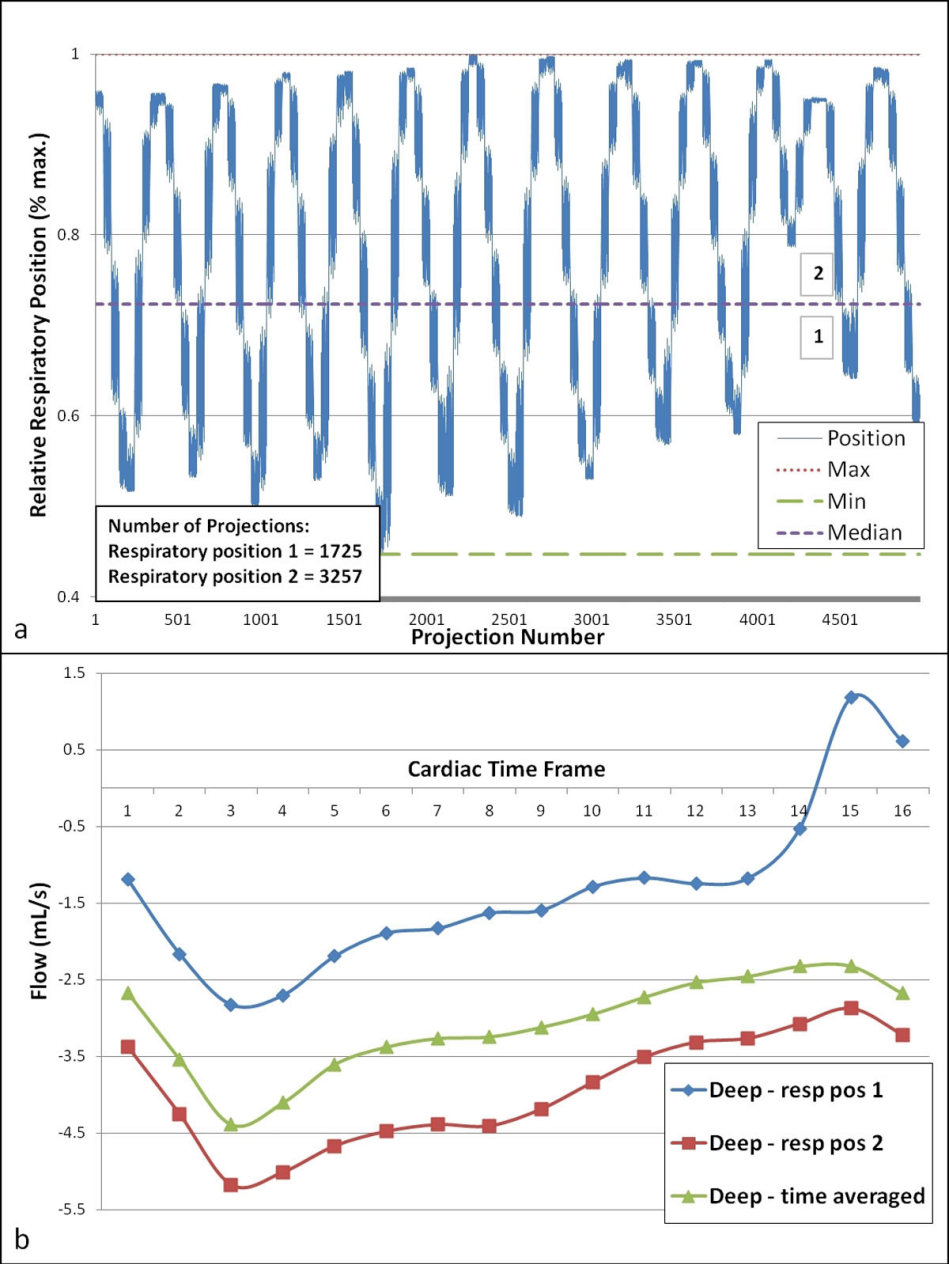
Example calculation of respiratory position widths for 2-position reconstruction (a). The respiratory waveform is broken into equal levels and projections are binned according to their position. Flow analysis shows increased magnitude flow during the “inspiration” plateau (position 2) versus position 1 (b). Time-averaged reconstruction gives flow roughly midway between position 1 and position 2.

Five volunteers were imaged on a clinical 3.0T system (Discovery MR750, GE) using a radially undersampled 2D PC sequence (4) prescribed axially at the level of the carotid bulb (scan parameters: FOV=24x24 cm, z=Δ5 mm, temporal resolution=77 ms, scan time=60 s, TR/TE=8.2/4.9 ms, α=15°, Venc=70 cm/s, projections≈5000). Respiratory waveforms recorded from bellows are used to sort the projections into respiratory phases in an offline reconstruction. Cardiac gating is achieved in a similar fashion based on the ECG signal, resulting in a double-retrospectively-gated PC-MR exam. The radial acquisition allows for reconstruction flexibility because each readout samples central k-space. Temporal view sharing is used for cardiac gating to improve the image quality reducing undersampling artifacts (5). The data can be grouped in arbitrary numbers of respiratory phases, thereby trading off image quality, scan time, and artifact level from radial undersampling. Data were acquired during regular and deep breathing and reconstructed with respiratory gating by grouping the data into 2 respiratory positions: around the inspiration plateau and the expiration plateau (Fig 1a). Flow analysis was performed in the internal jugular vein (IJV).

## Results

**Figure 2:**
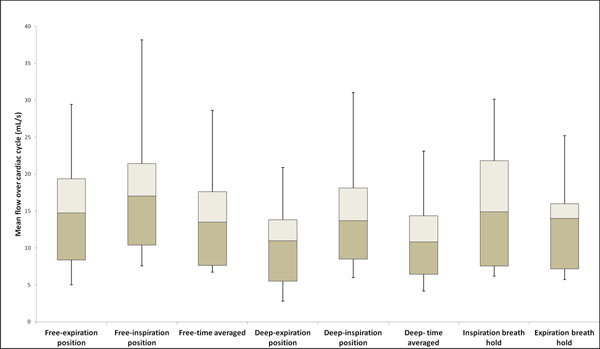
Mean flow over cardiac cycle for 5 volunteers in the dominant side IJV. While inspiration and expiration breath hold show minimal difference, the position 2 of both deep - and free - breathing show much higher mean flow than for position 1 indicating higher flow near the ‘inspiration plateau’. Likewise, the time averaged reconstruction gives mean flows somewhere in between positions 1 and 2.

Example results are shown in Fig 1b for deep-breathing. For each volunteer, sorting of the data into 2 respiratory positions showed a significant variation between expiration (pos 1) and inspiration (pos 2) as compared to a time-average image reconstructed without respiratory gating (Fig 2).

## Conclusions

Data acquired using the radial PC sequence with a double-gated reconstruction scheme confirms respiratory motion affects venous flow waveforms in the IJV. As known from ultrasound studies, the negative thoracic pressure during inspiration decreases resistance to flow in the IJV, thus increasing flow in venous drainage. Based on our initial results, we recommend that the influence of respiratory motion should be considered for quantitative venous flow measurements in the neck.
